# Intestinal Barrier Function in Gluten-Related Disorders

**DOI:** 10.3390/nu11102325

**Published:** 2019-10-01

**Authors:** Danielle Cardoso-Silva, Deborah Delbue, Alice Itzlinger, Renée Moerkens, Sebo Withoff, Federica Branchi, Michael Schumann

**Affiliations:** 1 Department of Gastroenterology, Rheumatology and Infectious diseases, Campus Benjamin Franklin, Charité—University Medicine, 12203 Berlin, Germany; 2 Department of Genetics, University of Groningen, University Medical Center Groningen, 9713GZ Groningen, The Netherlands

**Keywords:** epithelial barrier, permeability, celiac disease, non-celiac gluten sensitivity, non-celiac wheat sensitivity, wheat allergy

## Abstract

Gluten-related disorders include distinct disease entities, namely celiac disease, wheat-associated allergy and non-celiac gluten/wheat sensitivity. Despite having in common the contact of the gastrointestinal mucosa with components of wheat and other cereals as a causative factor, these clinical entities have distinct pathophysiological pathways. In celiac disease, a T-cell mediate immune reaction triggered by gluten ingestion is central in the pathogenesis of the enteropathy, while wheat allergy develops as a rapid immunoglobulin E- or non-immunoglobulin E-mediated immune response. In non-celiac wheat sensitivity, classical adaptive immune responses are not involved. Instead, recent research has revealed that an innate immune response to a yet-to-be-defined antigen, as well as the gut microbiota, are pivotal in the development in this disorder. Although impairment of the epithelial barrier has been described in all three clinical conditions, its role as a potential pathogenetic co-factor, specifically in celiac disease and non-celiac wheat sensitivity, is still a matter of investigation. This article gives a short overview of the mucosal barrier of the small intestine, summarizes the aspects of barrier dysfunction observed in all three gluten-related disorders and reviews literature data in favor of a primary involvement of the epithelial barrier in the development of celiac disease and non-celiac wheat sensitivity.

## 1. The Intestinal Barrier

**Figure 1:**
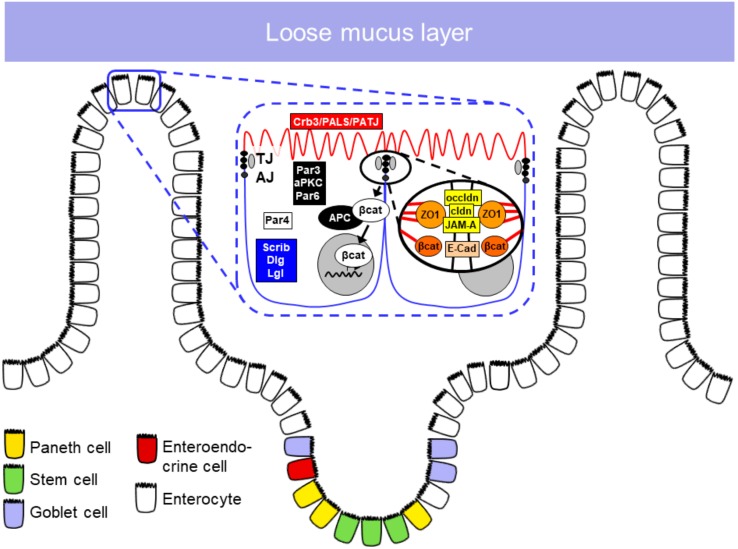
Mucus and epithelial barrier of the mucosa. TJ, tight junction; AJ, adherens junction; Crb3, crumbs3; PALS-1, protein associated with Lin7; PATJ, Pals1-associated tight junction protein; Par, Partition defective; aPKC, atypical protein kinase C; Scrib, scribble; Dlg, Discs large homolog-1; Lgl, Lethal giant larvae protein; βcat, β-catenin; APC, adenomatous polyposis coli protein; ZO1, zonula occludens protein-1; occldn, occludin; cldn, claudin; JAM-A, junctional adhesion molecule-A; E-Cad, e-cadherin.

The intestinal barrier has a crucial role in protecting the organism against pathogens and possible harmful substances derived from the external environment (Figure 1). It is formed by a mucus and epithelial layer and by the lamina propria underneath. Immune cells, components of the intestinal microbiota and anti-microbial peptides have crucial functions in maintaining the intestinal barrier function [1,2].

### 1.1. Mucus Barrier

In the gastrointestinal tract, the mucus barrier forms a protective layer covering the apical surface of the intestinal epithelium to avoid adherence and subsequent invasion by external pathogens [1]. Additionally, the mucus barrier lubricates food and digestive secretion, protecting the intestinal epithelium from possible damage [3,4].

#### Mucus Composition

Mucus is a complex viscoelastic adherent fluid produced and secreted by goblet cells. It is composed of approximately 95% water, which serves as a solvent and diffusion media for other molecules. One major component of mucus are mucins, highly glycosylated and very large proteins (Muc2, roughly 5200 amino acids) with regions rich in serine and threonine [5,6], which are pivotal for mucus generation as *MUC2* knockout mice do not form a mucus layer [7]. Complementary to that, lysozyme can also digest bacterial cell wall components [8,9]. Immunoglobulins, specifically secreted IgA, are associated to the mucus where they contribute in a complex fashion to the defense against pathogenic bacteria, regulate the mucus microbiota, and contribute to general mucosa homeostasis since lack of Igs leads to protein-losing enteropathy [10]. Growth factors such as transforming growth factor beta (TGFβ) are involved in growth, maintenance, repair and regulatory functions in the epithelium [11,12].

### 1.2. Epithelial Barrier

The intestinal epithelial barrier is the cellular covering of the intestinal wall. In this singly leveled cell layer cells are attached to each other by the apical junctional complex (i.e., the adherens junction and the tight junction), which at the same time seals the paracellular space to the intestinal lumen. Through cell division, maturation and cell migration, the epithelial cells are constantly renewed. The cell renewal in the small intestine occurs through stem cells present in the crypts. Different cell types develop from stem cells to compose the epithelial barrier, such as enterocytes, goblet cells, Paneth cells, microfold (M) cells and tuft cells [13,14,15].

#### 1.2.1. Epithelial Cell Types in the Small Intestine

The predominant cells are enterocytes, devoted to the absorption of nutrients, as well as to the protection of the epithelial surface protection by means of secretion of antimicrobial proteins [16]. Goblet cells are the main mucus-secreting cells that are—similarly to enterocytes—born in the crypt and then follow a migratory flow toward the surface epithelium but differentiate to a secretory cell type since they express the transcription factor Math1 [17]. Paneth cells play a crucial role in host defense against bacteria and regulation of the microbiota as they are major producers of α-defensins [18,19,20]. Moreover, they regulate epithelial renewal by nursing the stem cell compartment of the crypt [21]. M cells are a subset of epithelial cells highly specialized for antigen sampling. They transport antigens and intact microorganisms from the gut lumen to the lamina propria, in order to present them to immune cells and thus start the immune response [22]. Tuft cells monitor the intestinal lumen, and once there is an injury or bacterial infection, they transmit signals to immune cells in the underlying epithelia, activating the immune response [23].

#### 1.2.2. Apical Junctional Complex

In order to maintain the integrity of the intestinal epithelial barrier, epithelial cells are joined together by apical junctional protein complexes called tight junctions (TJ) and adherens junctions (AJ) (Table 1).

##### Tight Junctions

TJs are localized to the most apical part of the lateral epithelial cell membrane. Their main functions were previously described as being a (i) gate and at the same time (ii) a fence, in that the TJ barrier has the capacity to selectively control the luminal components that pass into the interepithelial space (gate function) and also to restrict lateral diffusion of membrane proteins as well as membrane lipids to either the apical or the basolateral compartment (fence function).

Main constituents of TJs include the transmembrane proteins occludin, the family of claudins and junctional adhesion molecules (JAM) [24]. Although occludin was the first TJ component to be identified, its role remains somewhat obscure. It was reported to be involved in the regulation of paracellular permeability, since loss of occludin affected the localization of tricellulin, a major constituent of the tricellular junction, and thus may affect the integrity of the epithelial barrier [25,26]. However, Saitou et al. [27] and Schulzke et al. [28] have shown that occludin knockout mice present normal TJ strand formation and normal barrier function. Furthermore, the hypothesis that occludin expression might define the epithelial leak pathway for macromolecules was not confirmed [29].

Together with occludin, claudins constitute the TJ strand and are the major components of this structure [30,31]. The family of claudins play a crucial role in TJ formation and the epithelial barrier, but they also have functions in cytoskeleton organization, transport of vesicles and signaling pathways directly associated with scaffold proteins such as ZO-1 [32]. Alterations in the expression of claudins are related to disturbance in homeostasis and contribute to several diseases [33,34,35]. The JAM family is localized in the TJ and it is composed, mainly, of three members: JAM-A, JAM-B and JAM-C. Among the member of the family, studies suggest that only JAM-A has a direct involvement in TJ maintenance. JAM-A was overexpressed in mouse fibroblast lacking TJ, however it failed to form strand-like TJ [36]. JAM-A may not have a direct role in the TJ formation. However, JAM-A deficient mice have shown a reduction in transepithelial electrical resistance, and an increased permeability for 4 kDa dextrans, suggesting a role for JAM-A in the regulation of paracellular permeability [37,38].

##### Adherens Junctions

The AJ is composed of two protein complexes associated with cell–cell adhesion: The nectin–afadin and the cadherin–catenin complex. These protein complexes have an extracellular region responsible for mediating adhesion of neighboring cells, while the intracellular component is involved in signaling, controlling of the AJ dynamic and interactions with the cytoskeleton. Cadherins are type I transmembrane proteins that interact calcium-dependently via their *N*-terminal domain with the identical protein of the adjacent cell. The intracellular, C-terminal domain interacts with β-catenin and connects thereby to the actomyosin network and EPLIN (epithelial protein lost in neoplasm). This complex is highly dynamic and modifies signaling via several pathways. Alterations in these pathways also contribute to tumor progression [39,40,41,42]. Nectins bind to afadin (AF-6, actin-binding protein) to form a structural adhesive complex directly linked to the cytoskeleton [24]. Ikeda et al. have shown that afadin knockout mice presented developmental defects with disorganization of AJ and TJ in the ectoderm during embryogenesis [43]. Sato et al. have shown that loss of afadin delays AJ formation, indicating that the nectin–afadin complex plays an important role in AJ maturation [44].

### 1.3. The Role of Lamina Propria Cells in Maintaining Barrier Function

The lamina propria is a supportive layer of conjunctive tissue and lies underneath the intestinal epithelium. Within this layer, immune cells, including macrophages, dendritic cells and lymphocytes play a crucial role in the defense against harmful substances and in maintaining the homeostasis of the intestinal epithelium [45].

#### 1.3.1. Dendritic Cells and Macrophages

Mononuclear phagocytes (macrophages and dendritic cells) are found in the gut-associated lymphoid tissue and in the intestinal lamina propria. These cells have various functions including phagocytosis for antigen sampling and/or clearance of pathogenic material as well as cytokine production and maintenance of epithelial barrier function [46]. Elegant work has shown that they have the capacity to sample antigenic material from the intestinal lumen by migration of cell protrusions through the intestinal epithelial layer. This occurs without altering barrier properties by dynamic expression of TJ proteins that interact with epithelial cells and thereby preserve the intestinal epithelia integrity [47,48]. Additionally, monocytes contribute to the maintenance of the epithelial barrier by producing the lipid mediator prostaglandin E2, which controls the neutrophil response to various microbiotic stimuli and thereby supports the homeostasis of the epithelial layer [49,50]. Macrophage subpopulations might even have regulatory potential as they express anti-inflammatory cytokines including interleukin-10 and thereby contribute to a regulatory, potentially tolerogenic immune response [51].

#### 1.3.2. Innate Lymphoid Cells

Innate lymphoid cells (ILCs) are innate immune cells belonging to the lymphoid lineage that were divided in three groups based on their cytokine and transcription factor expression profile, namely, ILC1, ILC2 and ILC3. ILC3 contribute to intestinal homeostasis inducing T-cell tolerance and to protection against intestinal infection by means of IL-22 secretion [52,53]. Nevertheless, ILC3 can also play a non-beneficial role in the gastrointestinal tract, as they have been associated with *Helicobacter hepaticus*-induced colitis, causing high levels of IL-17 and interferon-γ (IFNγ) [54]. Additionally, when producing extensive levels of IL-22, ILC3 might induce destruction of epithelia and consequently, intestinal damage [55].

### 1.4. Impact of Intraepithelial Lymphocytes on Barrier Function

Within the epithelial layer there is a population of T-cells known as intraepithelial lymphocytes (IELs). These cells, mostly CD3^+^ CD8^+^ T-cells, interact directly with enterocytes and are in close proximity to antigenic material in the gut lumen ready to initiate immune response [56,57]. Kuhn et al. have shown that interactions between commensal gut microorganisms and IELs promote the secretion of cytokines thereby enhancing epithelial barrier function [58]. Further, they have shown that loss of IL-6 reduced barrier function and TJ protein expression—a phenotype that was rescued by transfer of IL-6-expressing IELs. However, changes in the IEL population also contributes to deterioration of barrier function in inflammatory diseases. The presence of increased IELs is defined as a hallmark for celiac disease (CeD) and is well-established as a diagnostic criterion for pathologists [59,60]. In active and refractory CeD (RCD, a small CeD subgroup not responding to at least one year of GFD), IELs are activated by the upregulation of IL-15, which leads to epithelial cell destruction and subsequently barrier dysfunction [61,62,63].

### 1.5. Role of the Luminal Microbiota on Barrier Function

The intestinal microbiota is a complex system that includes specific bacteria classified at mutualistic inhabitants or commensal bacteria. These bacteria have been associated with several physiological functions improving the digestion, absorption, vitamin synthesis and protection against enteric bacteria overgrowth in the gut [64]. It has been shown that commensal bacteria exert a role in epithelial cell turnover and renovation in mice treated with dextran sodium sulfate, a well-established colitis model. Mice with depletion of commensal bacteria were shown to be more susceptible to mucosal injury. On the other hand, intestinal microbiota were identified as a decisive factor in the development of irritable bowel syndrome [65]. The pathophysiological mechanisms that regulate the balance of the gut microbiota are still far from clearly understood.

## 2. The Role of the Intestinal Barrier in Gluten-Related Disorders

Gluten-related disorders are a group of clinical entities that share dietary exposure to gluten (or gluten-containing products) as an etiologic factor [66]. Gluten-related disorders are classified into autoimmune (such as CeD, dermatitis herpetiformis, gluten ataxia), allergic (wheat allergy) and possibly immune-mediated (such as non-celiac gluten/wheat sensitivity).

Gluten is a structural protein complex contained in wheat, barley, rye and other related cereals. Gliadins are components of gluten and represent the class of proteins with the most relevant etiologic role in gluten-related disorders [67]. They are alcohol-soluble and rich in glutamine and proline residues that make them resistant to complete digestion in the gut. Gliadin partial digestion generates two peptides that have been extensively studied: The immunogenic peptide 33-mer (pp. 57–89), which initiates a strong response of the adaptive immune system to gluten [68], and the 25 AA peptide (pp. 31–55) that may directly induce IL-15 production in enterocytes and dendritic cells [69].

Gliadin exposure alone alters the barrier properties of intestinal epithelial cells. In Caco-2 cells, the addition of gliadin leads to a reduction in transepithelial electrical resistance (TEER) levels and an increase in permeability to 4 kDa dextran that are associated with reduced expression of occludin, ZO-1, and claudins-3 and -4 [70]. Drago et al. described that exposure of duodenal biopsies from celiac patients to pepsin-trypsin digested gliadin (PT gliadin) promotes a sustained secretion of zonulin, presumed to be a haptoglobin 2 precursor, and a significant decrease in TEER levels, whereas in non-celiac controls the zonulin concentration increased only transiently and TEER did not change significantly [71]. Lammers et al. uncovered that gliadin-induced zonulin secretion is a MyD88-dependent mechanism mediated by the CXCR3 receptor [72]. Moreover, gliadin pp. 31–43 peptide rapidly increased the number of IL-15+ cells in the lamina propria of CeD in-vitro organ cultures and consequently promoted the expression of COX-2, CD25 in lamina propria mononuclear cells and CD83 on dendritic cells, indicating its capability to activate the innate immune system in celiac patients [69]. Furthermore, in active CeD, pp. 31–43 induced enterocyte apoptosis significantly. All these effects were dependent of IL-15 and p. 38 MAP kinase [69].

### 2.1. The Intestinal Barrier in Celiac Disease

Celiac disease is an immune-mediated condition that affects the small intestine, causing diarrhea, abdominal pain and malabsorption and can also be associated with extraintestinal manifestations [73]. In susceptible individuals, peptides derived from ingested gluten cross the epithelial barrier and are deamidated by the tissue transglutaminase 2 enzyme (TG2) in the lamina propria, thereby increasing their affinity to the HLA-DQ2/DQ8 molecules on the membrane of antigen presenting cells [73]. This condition develops upon gluten ingestion in genetically predisposed individuals presenting the human leukocyte antigen (HLA)-DQ2 and/or HLA-DQ8 haplotypes [73]. These HLA haplotypes play a key role in promoting the immune response by presenting the immunogenic gliadin peptides to gluten-specific CD4+ T-cells. Once activated, the CD4+ T-cells secrete various cytokines, including IFNγ and IL-21 [74]. This implements an immune cascade leading to mucosal damage that ultimately causes intestinal villous atrophy, the histopathologic hallmark of CeD. The gluten-free diet (GFD), to date the only available treatment for CeD, prevents tissue exposure to gliadin: the absence of the antigen stimulating the immune cascade leads to an arrest of the immune response and subsequent mucosal healing [66].

The first evidence of impaired intestinal barrier function in CeD dates back to the 1970s, when intestinal permeability to different sugars was assessed by analyzing their excretion in the urine after intestinal absorption. Celiac patients with villous atrophy present an increased permeability to disaccharides and a decreased permeability to monosaccharides as evaluated in lactulose/mannitol, lactulose/L-rhamnose or cellobiose/mannitol ratios [75,76,77]. Moreover, a gluten challenge temporarily increased these ratios in CeD patients [68]. Such changes in permeability to sugars are directly related to the properties of the paracellular pathway, i.e., the TJ function. Accordingly, Schulzke et al. observed fewer TJ strands as well as abnormal and discontinuous strands in the jejunum of children with active CeD when compared to controls [78]. Children with CeD responding to a GFD present a significant recovery of the architecture of the TJ network, even though still presenting a smaller number of TJ strands [78]. Those findings correlate with the electrical properties of the intestinal epithelium measured by one-path impedance spectroscopy: A reduction of roughly 50% in epithelial resistance was reported in the same active CeD patients compared to controls, while GFD-responding patients presented a significant, yet partial, recovery [79]. Reims et al. observed lower epithelial resistance in CeD patients with partial villous atrophy, subtotal atrophy or on a GFD; nonetheless, only the difference between partial atrophy and controls reached significance [80]. In our study, a 50% reduction in epithelial resistance in active celiac adult patients was found [81] as well as a significant recovery of the epithelial resistance values after GFD [82]. Those results suggest that important changes in the function of the TJ occur during active disease that are mostly recovered by the GFD. However, the slight difference between treated patients and controls may point to a primary/genetic barrier-related difference in CeD patients. Furthermore, patients with RCD were found to present epithelial resistances comparable to active patients, with a reduction of 40% in comparison to controls [83], indicating the damaging effect of the inflammatory process in the TJ function.

Changes in mucosal electrical parameters and ultrastructure of TJs reflect molecular alterations as reported by Ciccocioppo et al. in both TJ and AJ proteins in CeD patients [84]. Changes in immunoprecipitation of ZO-1 with anti-phosphotyrosine and anti-occludin, but not in total ZO-1 or occludin, were found in active CeD patients. Such findings are in concordance with the immunofluorescence analysis, where a significant decrease in membrane bound ZO-1 and occludin was seen. Regarding AJ proteins, there was no significant difference in total content of β-catenin or E-cadherin. However, β-catenin was extensively phosphorylated in active CeD patients and this finding correlated with a decrease in its co-immunoprecipitation with E-cadherin [84]. Szakál et al. evaluated proximal and distal biopsies of the duodenum of children with CeD and found elevated content of claudin-2 and -3 in both sites in comparison to controls [85]. A further evaluation of TJ proteins both in protein content and localization was performed by our group and revealed increased pore-forming claudin-2 and -15 and decreased barrier-forming claudin-3, -5 and -7 as well as a decrease in occludin in CeD patients [81]. Similar to Ciccocioppo, we found reduced and membrane-displaced ZO-1. There was a strong membrane claudin-2 signal only in the crypts of CeD patients. Claudin-3 was reduced at the membrane, claudin-5 and -15 were only partially localized at the membrane, and claudin-7 showed a very diffuse localization pattern. No changes were seen in occludin or claudin-1 and -4 [81]. Interestingly, substantial changes in polarization proteins Partition defective-3 (Par-3) and protein phosphatase-1 (PP-1) were also observed in CeD patients, indicating a role for development of epithelial polarity in the barrier function of CeD small intestinal mucosae [82]. In addition, RCD patients, who present impaired barrier function similar to active patients, present even more claudin-2 protein content than active patients in the duodenal crypts. Conversely, there was no decrease in claudin-3 protein, but in claudin-4. Claudin-4 was also found to be displaced from the membrane only in RCD patients [83].

The inflammatory process and the immune response in CeD also influence the barrier properties of the epithelial cells. Exposure of Caco-2 cells to tumor necrosis factor-α (TNFα) and IFNγ promote decrease in ZO-1 protein and a reduction in the membrane localization of both ZO-1 and occludin, besides an increase in β-catenin phosphorylation and a decrease of its interaction with E-cadherin [84]. Transforming growth factor-β (TGFβ) also alters the capability of intestinal epithelial cells to polarize by inhibiting TJ assembly [81]. In addition, important barrier effects are exerted by the innate immunity, especially by IL-15 in CeD. As mentioned before, gliadin fragments increase IL-15 expression in the lamina propria and in this way mediate innate immunity inducing enterocyte cytolysis [69]. The connection between IL-15 effects and enterocyte cell death lies in the interaction between IELs and epithelial cells. To illustrate this, Hüe et al. reported that pp. 31–43 gliadin promotes a strong expression of major histocompatibility complex (MHC) class I chain-related protein A (MICA) in epithelial cells, through IL-15 [61]. MICA-expressing cells can become targets of IELs that express the NKG2D receptor. Accordingly, it was shown that NKG2D-expressing IELs from CeD patients were able to induce epithelial cell lysis, although there is evidence that the concomitant activation of other receptors is also important for this effect [61]. In RCD patient samples it was shown that IL-15 promotes IEL survival by Bcl-2, Bcl-Lx and Mcl-1 through the JAK3/STAT5 pathway, which could also explain at least partially the barrier dysfunction that is seen in RCD patients [67]. Relevant players in the alterations in barrier function in CeD are summarized in Table 2.

#### Evidence for a Primary Barrier Defect in Celiac Disease

In line with functional evidence, genetic studies associate several barrier-associated genes with CeD, suggesting a potential primary defect in intestinal barrier function. Genome-wide association studies (GWASs) revealed that 39 loci, besides the extensively studied HLA-DQ2 and HLA-DQ8 variants, are associated with CeD [86]. The prioritized genes from four of these loci were predicted to play a role in cell–cell adhesion, based on co-expression with other genes [87,88]. Functional data further emphasized this role for the associated genes *LPP*, *C1orf106* and *PTPRK*. The LPP protein colocalizes with E-cadherin and has a role in E-cadherin-dependent cell adhesion of epithelial cells [89]. C1orf106 is involved in maintaining adherens junction stability and C1orf106^-/-^ mice have reduced intestinal barrier integrity [90]. PTPRK, a receptor present at epithelial cell–cell junctions, dephosphorylates regulators of cell–cell adhesion, thereby promoting junction integrity [91]. *PARD3*, a gene coding for a substrate of PTPRK6, was also genetically associated with CeD by an earlier study that focused on genes involved in tight junction functioning [92]. However, this finding was not replicated in larger GWASs [86,93].

The genetic variants associated with CeD and linked to the genes mentioned above often do not map to protein-coding regions, but to intronic regions of the genes, making it difficult to infer the exact variant–gene interaction. Assessing whether these variants alter gene expression levels of proximal genes in the relevant tissue and conditions, in this case intestinal epithelial cells in the presence and absence of CeD-relevant cytokines, could further confirm the causal link between variant and affected gene.

Since the currently identified associated variants explain only part of the heritability of CeD, there might be other barrier-affecting variants with small effect sizes still to be detected. The most recent large GWAS [86] made use of the Immunochip, designed for densely genotype distinct loci previously associated with immune-mediated diseases. Although very valuable to explore the genetics of CeD, this chip is biased towards immune-related loci while barrier-related loci might be overlooked. Novel barrier-related variants could potentially be detected by using genome-wide arrays with larger sample sizes or different techniques with higher resolution, such as whole-genome sequencing.

### 2.2. The Intestinal Barrier in Wheat Allergy

Wheat allergy is a disorder characterized by immune activation where T helper cells type 2 (Th2) mediate immunoglobulin E (IgE) and non-IgE reactions after exposure (usually dietary) to wheat [94,95]. It is mainly observed in children and tends to improve or resolve at adult age. Symptoms can be mild, such as atopic dermatitis, asthma, vomiting, abdominal pain and diarrhea, or as severe as life-threating anaphylaxis. The diagnosis for this type of allergy can be difficult, because no validated test shows sufficient accuracy. The diagnosis is usually based on the patient history and the treatment is dietary avoidance [96].

In non-IgE reactions, the presence of wheat can lead to an immune response characterized by inflammation mediated by T-cells and eosinophils in the GI tract.

The defective barrier is discussed as a major driver in non-IgE-mediated allergies such as eosinophilic esophagitis or eosinophilic gastritis [97]. In contrast, IgE-dependent allergies are believed to be the result of a breach in oral tolerance causing Th2-biased immune dysregulation and secondarily B-cell-specific IgE production. More specifically, naïve T-cells are polarized to Th2 cells after presentation of specific antigens by dendritic cells, then producing IL-4 and IL-13. This induces B-cells to mature to Ag-specific IgE-producing plasma cells. IgE have high affinity to specific FcεRI receptors present on mucosal and skin mast cells and circulating basophils. These cells carry cytoplasmatic granules containing histamine. The ingestion of allergic proteins works as a trigger for degranulation of granula-carrying cells, thereby releasing histamine and other granule components. These constituents then affect barrier function [97,98,99].

Mast cells are important to maintain the intestinal homeostasis and are involved in the regulation of mucosal integrity and epithelial barrier activity by ion and water secretion and permeability [100]. In the setting of skin inflammation, mast cells seem to have a protective role for the epithelial barrier. Sehra et al. demonstrated that higher levels of protein translocation through the skin barrier were observed in mast cell-deficient mice [101]. Evidence suggests that mast cells play a role in influencing the barrier function during nematode infection. In this situation, *Trichinella spiralis* infection induces activation of Th2 cells and consequently triggers a critical mucosal mastocytosis and mucosal leakiness, which is important to the nematode expulsion.

Furthermore, increase in the intestinal epithelial permeability and decrease in TJ protein levels are observed during allergic responses [102]. Leukotrienes, lipid derivatives, are involved in IgE-dependent allergy reactions and inflammatory processes, and were reported to affect the barrier function. In particular, leukotriene B4 receptor 2 (BLT2) plays a pivotal role in skin barrier function through regulation of TJ arrangement and of inflammatory cytokine production [103,104].

For eosinophilic esophagitis as an example of a non-IgE-dependent allergy that also can be induced by wheat proteins, an IL-13-dependent downregulation of the AJ protein desmoglein-1 was shown to be responsible for the barrier defect in this disease entity [105]. Altogether, these data indicate that the epithelial barrier function is altered in allergic reactions, even though no specific gliadin-related effects have been investigated in the setting of wheat allergy.

### 2.3. Barrier Function in Non-Celiac Gluten/Wheat Sensitivity

Non-celiac gluten sensitivity or, more broadly, non-celiac wheat sensitivity (NCWS) is a disorder characterized by the onset of gastrointestinal as well as extraintestinal symptoms such as tiredness, “foggy mind”, headache and anxiety following ingestion of gluten-containing food in individuals in whom CeD and wheat allergy have been excluded [66,106]. Its most common symptoms are abdominal pain, diarrhea and bloating that resolve rapidly after adhering to a gluten-free diet and reappear when challenged with gluten [66]. The difficulty in diagnosing NCWS lies in the lack of pathognomonic markers. Patients do not present villous atrophy nor produce specific antibodies or IgE in response to wheat ingestion [106]. Antibodies against native gliadin (AGA), were sometimes associated with NCWS, but have insufficient diagnostic accuracy [107]. The genetic marker HLA-DQ2 is not associated with this disease [108]. Exposure to food components other than gluten are currently under investigation as a possible etiologic factor for NCWS, such as α-amylase trypsin inhibitors (ATIs) [109] and fermentable oligo-, di- and mono-saccharides and polyols (FODMAPs) [110]. More recently, a pre-existing dysbiosis of the intestinal microbiome leading to a decrease in butyrate and altering the gut barrier against inflammatory stimuli was described [111].

Conflicting data exist on barrier function in NCWS. In the first study published on that topic, the lactulose/mannitol ratio was significantly reduced when compared to CeD patients and controls, i.e., the paracellular permeability appeared to be decreased when compared to CeD as well as dyspeptic control individuals. In concordance with the permeability findings, NCWS patients presented a higher RNA expression of claudin-4, a barrier-forming claudin, whereas no changes were found for claudin-1 to -3, ZO-1 and occludin [112]. On the contrary, Hollon et al. evaluated changes in TEER after exposure to PT-gliadin in tissue biopsies from CeD, NCWS patients and non-celiac controls. In this study, exposure to gliadin reduced TEER in all groups. However, significant changes were observed when comparing active celiac or NCWS patients to celiac patients in remission, indicating that although severe mucosal alterations are not seen in NCWS patients, there is a barrier-impairing effect exerted by PT-gliadin in NCWS similarly to CeD [113]. Confirming the conclusion that barrier function is rather reduced in NCWS, Fritscher-Ravens et al. published work on in vivo analyzed human barrier function in NCWS by using confocal endomicroscopy after intravenous injection of fluorescein. Here, small intestinal epithelial defects and luminal leakage of fluorescein occurred in NCWS patients only minutes after luminal exposure to wheat and were associated with increased expression of the pore-forming claudin-2 [114,115]. Notably, an impaired epithelial barrier function could contribute to NCWS by altering the interaction between the intestinal microbiota and the systemic circulation. Uhde et al. observed increased levels of lipopolysaccharide-binding protein and sCD14 in NCWS patients, indicating increased translocation of microbial products and increased EndoCAb IgM and anti-flagellin antibodies. These findings suggest that microbial translocation triggered both innate and adaptive immune responses [116]. In addition, there was a positive correlation between those findings and epithelial damage as assessed by the circulating levels of FABP2. Finally, there was significant reduction in all cited molecules in response to dietary ablation of wheat, rye and barley. These results reinforce the hypothesis that impairment in the intestinal epithelial barrier leads to increased microbial translocation and contributes to the pathophysiology of NCWS.

## 3. The Role of Barrier Function Tests in Assessing Intestinal Permeability

To date, there are several available diagnostic tools to assess gut permeability and therefore indirectly measure intestinal barrier function. Their validity and applicability in the clinical setting, especially for the diagnosis of gluten-related disorders, is yet to be clarified.

### 3.1. In Vivo Tests

One established method of assessing gut permeability is to orally administer a tracer molecule, such as the non-digestible sugars lactulose or mannitol (cited above), fluorescently labeled dextrans, PEG and ^51^Cr-EDTA, and to subsequently detect and quantify those tracers in the blood or urine. Depending on the size of the used tracer the subcellular localization of the intestinal leak can be assessed further. While mannitol, ^51^Cr-EDTA and 4 kDa dextrans use the paracellular route, molecules like 40 kDa HRP pass via the transcellular route [117,118]. A limitation to this diagnostic tool is that the affected gastrointestinal region cannot be distinguished and other factors such as gastric emptying, bacterial flora, and intestinal blood flow have to be considered as they have a significant influence on the tracer’s distribution and excretion [119]. Moreover, the practical application of this method poses difficulties in the clinical routine, so that their use is mostly limited to research settings [71,120,121].

### 3.2. Ex Vivo Analysis of Barrier Function

Mucosal explants of human small intestinal tissue (collected by small bowel endoscopy) can be mounted to modified Ussing chambers. Various parameters can be analyzed under physiological conditions (regarding pH and temperature) including the short-circuit current (I_sc_) to analyze epithelial ion transport and electrical resistance. Conditions can be altered specifically on the mucosal or serosal side of the tissue explant. Moreover, one-path impedance spectroscopy can be applied that discriminates vertically between two serial pathways, namely, the resistances of the epithelial cell layer and of subepithelial tissues. Another option is to measure macromolecular fluxes revealing, for example, a dextran permeability that complements the resistance measurements that reflect conductance for small ions [122,123]. Another, more recently developed option to study barrier function in ex vivo mucosae is the sandwich assay, allowing for local resolution of macromolecular permeability using an avidin/biotin-based high resolution microscopy approach [124,125]. This method is able to determine the paracellular macromolecule passage in epithelial sheets, thus helping in the discrimination between barrier defects.

### 3.3. Non-Invasive Biomarkers

There are a number of known biomarkers found in urine, blood or feces that can be used as non-invasive markers of intestinal permeability. Evidently, their advantage lies in their non-invasiveness. There is no need for oral or intravenous administration of marker molecules. Their downsides are the generally lower sensitivity and specificity. Claudins can be detected in urine samples; urinary claudin concentrations may reflect the intestinal barrier function. Alternatively, fatty acid binding protein (FABP) and glutathione s-transferase (GSTs) levels can be traced in the urine or even be detected in serum or plasma—both are released from the intestinal cell membrane upon epithelial damage [126]. Citrulline—a non- proteinogenic amino acid—has also been described as a potential marker for intestinal barrier function. Recent studies have established that citrulline concentration correlates with extent of intestinal failure [127]. A quantitative sandwich enzyme-linked immunosorbent assay (ELISA) has been established to quantify the serum levels of zonulin, a player in the intestinal response upon gliadin exposure, as a biomarker of the individual intestinal barrier function. Moreover, since studies suggested that carriers of the *HP2* allele (zonulin gene) are at a higher risk of developing inflammatory diseases, genotyping via RT-PCR has been suggested as a diagnostic tool to assess the risk of autoimmune diseases [107]. However, a recent study found significant fluctuations in zonulin levels in patient groups with gastrointestinal conditions compared with healthy individuals and unveiled major inconsistencies in the detection of the applied assays [82,121]. Overall, currently available non-invasive assays lack diagnostic accuracy to sufficiently reflect the individual barrier function. However, a combination of in vivo and ex vivo tests of permeability as done in Ussing chambers can image the individual´s barrier function [118].

In perspective, new methods for assessing the gut barrier will be established. Fritscher-Ravens et al. used confocal laser endomicroscopy (CLE) to examine patients revealing an irritable bowel syndrome-like phenotype. Diluted food antigens were applied directly on the duodenal mucosa through the endoscope and fluorescein was infused intravenously. CLE was performed to identify epithelial leaks for fluorescein. Fluorescein permeabilities were analyzed before and after exposure of the mucosa to various food antigens. The study revealed the potential of this technique to identify patients with rapidly occurring barrier defects. Thus, CLE represents an interesting, somewhat labor-intensive diagnostic tool which may become practicable in the future [114].

## 4. Is the Barrier a Potential Therapeutic Target?

In view of the increasing evidence consistent with a direct role of the intestinal barrier in the pathogenesis of gluten-related disorders, particularly as it concerns CeD, efforts have been made to identify components of the epithelial barrier as possible therapeutic targets.

So far, the therapeutic target within the intestinal epithelial barrier most extensively studied is zonulin [128]. As mentioned above, gliadin can activate zonulin signalling and alter intestinal permeability [71,129]. An inhibitor of zonulin, larazotide acetate (originally AT1001), was tested for its ability to reverse the increased gut permeability caused by zonulin signalling [129,130]. In vitro experiments showed that larazotide acetate was able to inhibit TJ rearrangements, thus preventing a higher epithelial permeability triggered by exposure to gliadin, cholera-like toxin and proinflammatory cytokines [129].

In view of promising preliminary results, larazotide acetate has been further tested in Phase I and II clinical trials. A first study on 20 CeD patients who underwent a three-day course of larazotide versus placebo followed by an oral gluten challenge demonstrated an increase in gut permeability after the gluten challenge in the placebo group, but not in the larazotide group [130]. Two subsequent studies on 86 and 184 CeD patients were not able to entirely replicate those results [131,132]. In the study by Leffler et al., treatment with larazotide acetate was administered for 14 days at different doses as compared to placebo, while in the study by Kelly et al. CeD patients received a daily gluten challenge (2.7 g gluten) for six weeks along with larazotide or placebo. In both studies, no significant differences in intestinal permeability were observed between groups as measured by lactulose/mannitol extraction fraction. However, symptom improvement was observed in patients taking larazotide as compared to placebo. Moreover, Kelly et al. found higher levels of anti-TG antibodies in the placebo group, suggesting a role in larazotide in preventing the activation of the immune response to gluten. The lack of significant differences in gut permeability between groups was interpreted as possibly consequent to intrinsic variability of urinary lactulose and mannitol excretion [131]. A different approach was adopted in a further trial, where larazotide (at the doses of 0.5, 1 or 2 mg per day) was tested against placebo in a large group of 342 celiac patients with persistent symptoms despite a correct GFD [133]. In that study the primary endpoint were gastrointestinal symptoms, which improved significantly in the 0.5 mg larazotide treatment group as compared to placebo, but not in the other dosage groups.

The actual therapeutic effect of larazotide in CeD so far remains controversial. The significant difference in gut permeability in vivo between larazotide and placebo, as measured by the mannitol/lactulose gradient in the first study, could not be replicated in further trials, despite observations of differences in gastrointestinal symptoms. Before larazotide can be introduced as a treatment option in CeD (and possibly in NCWS), more data are needed to corroborate conflicting evidence and clarify uncertainties about its pharmacodynamics. An ongoing Phase 3 trial (NCT03569007) is testing lower doses of larazotide on patients on GFD, in view of the observation in the previous studies that lower doses of larazotide had a stronger effect in reducing gastrointestinal symptoms, which was interpreted as possibly due to reduced activity at higher doses because of peptide aggregation [133].

To date, no other molecules with an effect on intestinal barrier function have been tested as potential therapeutic options for CeD or gluten-related disorders.

## 5. Conclusions

Although CeD is regarded as a T-cell disease involving gliadin-specific T-cells, recent research has revealed a role for barrier function also in the induction phase of the disease. Hypothetically, barrier disturbances might account for the variability of CeD induction in the course of the patient´s life—in many individuals CeD is induced in early childhood. However, in a considerable number of CeD cases disease activity was only noted at an age >30 years. Transient loss in barrier function, e.g., after bacterial or viral enteral infections, might account for this phenomenon. Similar to other chronic inflammatory bowel diseases such as Crohn´s disease or ulcerative colitis, where the interaction of microbiota and mucosal barrier plays a decisive role in immunopathology, currently available diagnostics lack the potential to easily and rapidly determine the patient´s barrier function. However, new methods are on the rise, which could potentially improve knowledge about the role of the epithelial barrier in the pathogenesis of CeD. Moreover, the first clinical studies to specifically target barrier function as a treatment option in CeD have been undertaken.

## Figures and Tables

**Table 1:** Components and functions of the apical junctional complex.

**Tight Junction**	**Functions**
Occludin	Constitution of TJ strand?
Claudins	TJ and epithelial barrier formation Constitution of TJ strands Cytoskeleton organization
JAM	TJ maintenance Role in barrier regulation
**Adherens junction**	**Functions**
Nectin-afadin	AJ organization and maturation
E-cadherin-β-catenin	Interaction with components of the cytoskeleton Organization and maintenance of AJ

TJ, tight junctions; JAM, junctional adhesion molecule; AJ, adherens junctions.

**Table 2:** Barrier impairment findings in celiac disease.

Type of Change	Observations	Reference
Functional	Increased cellobiose/mannitol excretion ratio	[75]
Functional	Increased Lactulose/L-rhamnose excretion ratio	[76]
Functional	5-fold increase in Lactulose/mannitol excretion ratio	[77]
Functional	13-fold increase in cellobiose/mannitol ratios in active CeD. 2-fold increase in treated patients and 5-fold increase in non-responders	[68]
	Cellobiose/mannitol excretion ratio reached normal levels after treatment and increased transiently after a gluten challenge	
Functional	56% reduction in electrical epithelial resistance in active CeD and 25% reduction in treated CeD	[79]
Structural	Decreased number of tight junction strands and depth of TJ meshwork in active CeD. Partial recovery in treated CeD	[78]
	Discontinued strands and aberrant strands below the main junctional meshwork	
Molecular	Loss of co-immunoprecipitation of occludin and ZO-1 despite no changes in total protein levels. Decrease in the membrane localization of ZO-1 and occludin	[84]
	Loss of co-immunoprecipitation of E-cadherin and β-Catenin despite normal levels of in total protein. Extensive phosphorylation of β-Catenin. Redistribution of both AJ proteins from the membrane to the cytoplasm	
Functional	26% reduction in paracellular resistance in CeD patients with partial and subtotal atrophy. 16% reduction in treated CeD	[80]
Functional	Electrical resistance decrease after exposure to PT-gliadin in samples from CeD patients	[71]
Molecular	Zonulin release and decrease in occludin gene expression after PT-gliadin exposure	
Molecular	Increased claudin-2 and -3 in patient samples with villous atrophy	[85]
Molecular	Increased claudin-2, -15 and decreased claudin-3, -5, -7 and occludin. No changes in claudin-2 RNA. Intense claudin-2 staining in the TJ of the crypts. Reduced membrane localization of claudin-3 and ZO-1. Partial membrane localization of claudin 5 and 15 and inhomogeneous claudin-7 staining	[81]
Functional	48% reduction of electrical resistance in CeD	
Molecular	Increased Claudin-2 in the crypts and decreased Claudin-4 and -5 in Refractory patients	[83]
Functional	40% decrease in epithelial resistance in refractory patients	
Functional	Only partial recovery of epithelial resistance in treated CeD	[82]

CeD, celiac disease, TJ, tight junction, AJ, adherent junction.
